# Review on the Cellular Mechanisms of Low-Level Laser Therapy Use in Oncology

**DOI:** 10.3389/fonc.2020.01255

**Published:** 2020-07-24

**Authors:** Shing Yau Tam, Victor C. W. Tam, Shanmugasundaram Ramkumar, May Ling Khaw, Helen K. W. Law, Shara W. Y. Lee

**Affiliations:** ^1^Department of Health Technology and Informatics, Faculty of Health and Social Sciences, The Hong Kong Polytechnic University, Kowloon, Hong Kong; ^2^Department of Clinical Oncology, NHS Foundation Trust, University Hospital Southampton, Southampton, United Kingdom; ^3^Tasmanian School of Medicine, University of Tasmania, Hobart, TAS, Australia

**Keywords:** cellular mechanism, inflammation, low-level laser therapy, metabolism, oncology, oncology treatment side effects, photobiomodulation

## Abstract

Photobiomodulation (PBM) using low-level laser therapy (LLLT) is a treatment that is increasingly used in oncology. Studies reported enhancement of wound healing with reduction in pain, tissue swelling and inflammatory conditions such as radiation dermatitis, oral mucositis, and lymphedema. However, factors such as wavelength, energy density and irradiation frequency influence the cellular mechanisms of LLLT. Moreover, the effects of LLLT vary according to cell types. Thus, controversy arose as a result of poor clinical response reported in some studies that may have used inadequately planned treatment protocols. Since LLLT may enhance tumor cell proliferation, these will also need to be considered before clinical use. This review aims to summarize the current knowledge of the cellular mechanisms of LLLT by considering its effects on cell proliferation, metabolism, angiogenesis, apoptosis and inflammation. With a better understanding of the cellular mechanisms, bridging findings from laboratory studies to clinical application can be improved.

## Introduction

Photobiomodulation (PBM) describes the changes in cellular activity and transformation in response to irradiation with light under certain conditions. Phototherapy with ultraviolet light has been used for many years in the treatment of psoriasis (1) or neonatal jaundice (2). Recently, with wider availability of instruments, PBM using low-level laser therapy (LLLT) has provided an exciting new frontier in the management of wound healing, pain, tissue swelling and particularly for oncology, the inflammatory conditions such as radiation dermatitis (RD), oral mucositis (OM) and lymphedema (LE) (3, 4).

RD and OM are well documented complications of radiotherapy (RT) for which management had been limited to only supportive treatments in the past. LE is caused by disruption to the lymphatics as a result of lymph node dissection during surgery or RT in breast or head and neck cancer patients (3). Recent studies suggest that PBM using LLLT is effective in preventing or mitigating these complications by preconditioning the cells to reduce inflammation and promote tissue healing (5, 6). This is a relatively novel treatment modality for which the cellular mechanisms are poorly understood, but is crucial for consideration before its routine application in oncology patients.

Several laboratory studies have reported the effects of LLLT on cell proliferation, metabolism, angiogenesis, apoptosis, and inflammation. Unlike pharmaceutical agents, LLLT involves a wide range of parameters in terms of laser properties and dosage which has been shown to be important for the effects to occur (7). Underdosage results in poor cellular response but overdosage may paradoxically inhibit cell proliferation or induce apoptosis. These cellular responses also appear to be specific for the tissue type. Moreover, Hamblin et al. (8) have reported that these cellular responses were also observed in some types of tumor cells that were irradiated, implying that tumor growth may possibly be enhanced. Thus, it is important that the cellular effects of LLLT are better understood and considered before the formulation of clinical treatment protocols for its use in oncology patients.

This review aims to summarize the current evidence for the effects and mechanisms of LLLT at cellular level. Particularly, the effects of LLLT induced changes in normal and tumor cells will be discussed, so that the transition from laboratory findings to clinical practice can be safely implemented for oncology patients.

## Influence of LLLT at Cellular Level

The findings of various studies that have investigated the influence of PBM at cellular and molecular levels are summarized in Table 1 and discussed below.

**Table 1:** Cellular and molecular studies of the influence of photobiomodulation.

**Cell type**	**Light source**	**Wavelength**	**Treatment regimen**	**Results**	**Reference**
Fibroblast (Human periodontal ligament)	Laser	809 nm	1.96–7.84 J/cm^2^, 1–3 treatments	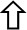 Proliferation	(9)
Fibroblast (Human periodontal ligament)	Laser	660 nm	350 mW, 15 min daily treatment X 3 days	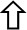 Proliferation	(10)
Fibroblast (NIH-3T3)	Laser	632.8 nm	2.5, 5.0, 16.0 J/cm^2^, 1–3 treatments X 2 days	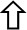 Proliferation, migration (2.5 J/cm^2^, 2–3 daily treatments, 5.0 J/cm^2^, 1 daily treatment), Proliferation, migration, viability and ATP activity (16.0 J/cm^2^)	(11)
Fibroblast (NIH-3T3)	Laser	904 nm	3, 4, 5 J/cm^2^, daily treatment X 1–6 days	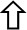 Proliferation (3, 4 J/cm^2^)	(12)
Fibroblast (HFF-1)	Laser	660 nm	3–8 J/cm^2^, ST	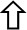 Proliferation, anti-inflammatory cytokine (IL-10) (Best dose at 4 J/cm^2^)	(13)
Fibroblast (Human dermal)	Laser	830, 980, 2,940 nm	5.5 J/cm^2^, ST	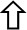 Proliferation (830, 980 nm), Proliferation, Apoptosis (2,940 nm)	(14)
Fibroblast (Human dermal)	Laser	635, 830, 635 + 830 nm	60 J/cm^2^, ST	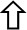 Proliferation, collagen synthesis (830, 635 + 830 nm)	(15)
Fibroblast (Human reticular and papillary dermal)	LED	450, 490, 550, 590, 650, 850 nm	0–250 J/cm^2^, ST	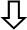 Metabolism (450, 490 nm)	(16)
Keratinocyte (Human)	Laser	780 nm	0.5–7 J/cm^2^, daily treatment X 3 days	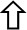 Proliferation, type I collagen, VEGF (All energy densities)	(17)
Stem cell (Human dental pulp)	Laser	660 nm	3 J/cm^2^, 20 mW/6 s and 40 mW/3 s	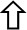 Proliferation (20 mW/6 s)	(18)
Stem cells (Mesenchymal, cardiac)	Laser	804 nm	1, 3 J/cm^2^, ST	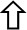 Proliferation (1, 3 J/cm^2^)	(19)
Stem cell (Human exfoliated deciduous teeth)	Laser	660 nm	2.5 J/cm^2^, 1–3 treatments; 5.0 and 7.5 J/cm^2^, ST	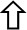 Proliferation (2.5 J/cm^2^, 3 treatments, 5.0 and 7.5 J/cm^2^)	(20)
Osteoblast	Laser	685 nm	2 J/cm^2^, 1–2 treatments	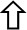 Proliferation, viability, bFGF, IGF-I, IGFBP3	(21)
Stem cell (Bone marrow-derived mesenchymal)	Laser	810 nm	2–6 J/cm^2^, 3 treatments in every other day	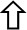 Differentiation, proliferation (2–4 J/cm^2^)	(22)
Osteoblast	Laser	632 nm	0.14–1.43 J/cm^2^, daily treatment X 2 days	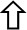 Differentiation, proliferation (All energy densities)	(23)
Fibroblast (HFFF-2)	Laser	647 nm	1.5 mJ/cm^2^, ST	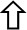 Mitochondrial membrane potential, intracellular calcium, ROS production, cytoplasmic alkalinization	(24)
Osteoblast (OFCOL-II)	Laser	830 nm	3 J/cm^2^, daily treatment X 3 days	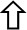 Proliferation, change of mitochondrial morphology from filamentous to granular appearance	(25)
Fibroblast (Murine embryonic)	Laser	810 nm	0.003–30 J/cm^2^, ST	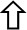 ROS, ATP production, NF-κB activation	(26)
Cardiomyocyte	Filtered light	400–800 nm	3.6, 12 J/cm^2^, ST	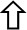 ROS production	(27)
Fibroblast (Human dermal, injured)	Laser	632.8 nm	5, 16 J/cm^2^, 2 treatments (Day 1 and 4)	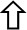 Mitochondrial membrane potential, intracellular calcium, ATP, cAMP (5 J/cm^2^), ATP, cAMP (16 J/cm^2^)	(28)
Neutrophil polymorphonuclear granulocyte, keratinocyte	Laser	660, 800, 970, 660 + 800 + 970 nm	3–6 J/cm^2^, ST	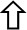 ROS production (660 nm), ROS production (800, 660 + 800 + 970 nm)	(29)
Neuron (Dorsal root ganglion)	Laser	800, 970 nm	6 J/cm^2^, ST	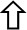 Mitochondrial membrane potential, ROS production (800 nm), Calcium flow (970 nm)	(30)
Endothelial cell (HUVEC)	Laser	660, 780 nm	1–20 J/cm^2^, ST	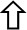 Cell viability, protein concentration (660 nm), 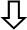 Cell viability (780 nm)	(31)
Granulosa cell (KGN)	Laser	830 nm	60 mW/60 s, ST	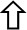 VEGF, MAPK	(32)
Thoracic aortal ring (Sabra rat)	Laser	660 nm	20 mW/10 min, ST	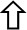 Angiogenesis	(33)
Endothelial cell (HUVEC)	LED	635 nm	24 J/cm^2^, ST	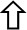 Proliferation, cellular network formation	(34)
Fibroblast (Human keloid, Murine NIH-3T3)	Laser	660 nm	150, 1,050 J/cm^2^, daily treatment X 3 days	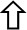 Proliferation (Human keloid, 150 J/cm^2^), 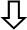 Proliferation (Human keloid, 1,050 J/cm^2^), 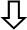 Cell death (150 J/cm^2^), 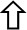 Hypodiploid cell (1,050 J/cm^2^)	(35)
Monocyte/macrophage (Mouse, RAW 264.7)	Laser	660 and 808 nm	11–214 J/cm^2^, ST	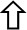 NO release (660 nm, 64 and 214 J/cm^2^)	(36)
M1 and M2a macrophage (Mouse, J774)	Laser	660 and 780 nm	17.5 J/cm^2^, ST	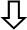 CCL3, CXCL2, TNF-α (660 nm, M1, 4 h), 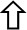 CXCL2, TNF-α (660 nm, M1, 24 h), 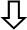 CCL3, IL-6 (780 nm, M1, 24 h), 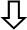 CXCL2 (660 nm, M2a, 4 h), 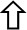 TGF-β1 (780 nm, M2a, 4 h), 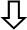 TGF-β1 (780 nm, M2a, 24 h)	(37)
M1 macrophage (Mouse, J774)	Laser	660 and 780 nm	2.6, 7.5 J/cm^2^, 1–2 treatments	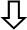 TNF-α, iNOS, COX-2 (660 and 780 nm), 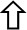 IL-6 (660 nm)	(38)
Endothelial cell (Human)	Laser	660 nm	11.46 J/cm^2^, ST	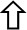 Proliferation, 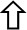 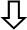 TNF-α/cycloheximide-induced apoptosis, caspase-3/7/8/9	(39)
Fibroblast (Human periodontal ligament)	Laser	660 nm	8 J/cm^2^, ST	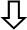 LPS-induced pro-inflammatory cytokines	(40)
Stem cell (Human umbilical cord mesenchymal)	Laser	635, 808, 635 + 808 nm	12 J/cm^2^, 2 daily treatments X 3 days	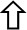 Pro-inflammatory cytokines (IL-1, IL-6), NF-κB, proliferation, ROS production	(41)

*ATP, Adenosine triphosphate; bFGF, basic fibroblast growth factor; cAMP, Cyclic adenosine monophosphate; CCL3, Chemokine (C-C motif) ligand 3; CXCL2, Chemokine (C-X-C motif) ligand 2; IGF-I, Insulin-like growth factor-I; IGFBP3, Receptor of IGF-I; IL, Interleukin; iNOS, Inducible nitric oxide synthase; LED, Light emitting diode; LPS, Lipo-polysaccharide; MAPK, Mitogen-activated protein kinase; NF-κB, Nuclear factor-κB; NO, Nitric oxide; ROS, Reactive oxygen species; ST, Single treatment; VEGF, Vascular endothelial growth factor*.

### Proliferation and Differentiation

Since wound healing is enhanced, it is logical that laboratory studies were directed at exploring the effects of LLLT at cellular level on cell proliferation.

Fibroblasts are the main type of cells involved in healing following tissue damage and are the most frequently studied. Kreisler et al. (9) evaluated the impact of GaAlAs diode laser (809 nm, 1.96–7.84 J/cm^2^, 1–3 treatments) on the proliferation of human periodontal ligament fibroblasts by fluorescence activity of a REDOX indicator (Alamar Blue Assay), and found significant increase of cell proliferation up to 72 h after LLLT. Schartinger et al. (10) reported similar results, with LLLT-treated (660 nm, 350 mW, three 15 min daily exposures) fibroblasts having significantly higher cell proliferation level than controls using MTT (3-(4,5-dimethylthiazol-2-yl)-2,5-diphenyl tetrazolium bromide) assay (0.37 ± 0.11 vs. 0.23 ± 0.10, *p* < 0.001).

Some studies reported more complex responses of fibroblasts under different energy density or wavelength of LLLT. Hawkins and Abrahamse (11) studied the responses of wounded human skin fibroblasts to various doses of HeNe (632.8 nm) laser treatment that employed 2.5, 5.0, and 16.0 J/cm^2^, 1–3 treatments daily for two consecutive days. The results showed that 2.5 J/cm^2^ of 2–3 daily treatments and 5.0 J/cm^2^ of single daily treatment increased cell proliferation and migration, with cell viability maintained without stress or cell damage. However, exposures at 16.0 J/cm^2^ inhibited cell proliferation with negative impacts on cell migration, viability and adenosine triphosphate (ATP) activity. Another study also demonstrated similar findings that Ga-As diode laser (904 nm) irradiation of 3 or 4 J/cm^2^ over a period of 1–6 days increased NIH-3T3 fibroblast cell numbers by 3–6 times compared to control. However, a high energy density using 5 J/cm^2^ did not stimulate cell growth (12). Similar results were found in other studies (13) to confirm the bio-stimulatory effects of LLLT at a limited energy density range and excessively high energy density may result in the opposite effect.

Apart from energy density, studies also focused on the influence of different wavelength on fibroblast proliferation. Crisan et al. (14) compared the effects of 830, 980, and 2,940 nm lasers (5.5 J/cm^2^) on human skin fibroblasts by MTT assay and apoptosis assay. They demonstrated that both 830 and 980 nm significantly stimulated cell proliferation at 24, 48, 72 h post-irradiation but 2,940 nm inhibited cell proliferation and promoted apoptosis. Ma et al. (15) reported increased human fibroblast proliferation and collagen synthesis by 830 or 635 nm and 830 nm dual wavelength (60 J/cm^2^) LLLT, but 635 nm alone did not produce significant fibroblast proliferation or collagen synthesis. Mignon et al. (16) reported that a short-wavelength of light (<530 nm) inhibited the metabolic activity of human dermal fibroblasts, but not with longer wavelengths of 550–850 nm. These studies suggest that the bio-stimulatory effects of only a specific range of wavelength is effective.

The interactions between keratinocytes and fibroblast also plays a crucial role in wound healing (42). Basso et al. (17) evaluated the effects of LLLT using an InGaAsP diode laser (780 nm, 0.5, 1.5, 3, 5, and 7 J/cm^2^, 3 times daily). Using MTT assay, all energy densities showed an improvement in cell proliferation, with better responses at 0.5–3 J/cm^2^. There were also improvements in type I collagen and vascular endothelial growth factor (VEGF) gene expression after LLLT.

Another commonly studied cell type is stem cell, which is an important component in tissue regeneration. A study investigated the impact of LLLT from InGaAlP laser (660 nm) with two power settings (20 mW/6 s and 40 mW/3 s) at 3 J/cm^2^ on human dental pulp stem cells (18). Results found that 20 mW had significantly higher cell proliferation than control or 40 mW groups. Post-LLLT cells cultured under nutritional deficit medium had significantly higher cell viability than non-irradiated cells. LLLT (Ga-As laser, 804 nm, 1 and 3 J/cm^2^) treated mesenchymal stem and cardiac stem cells had increased cell proliferation up to 4 and 2 weeks post-LLLT, respectively, and there were no differences between the two energy densities (19). Stem cells from human exfoliated deciduous teeth treated with LLLT (660 nm, 2.5–7.5 J/cm^2^, 1–3 irradiations) also had similar cell viability and stimulated cell proliferation at 72 h (20). The effect of LLLT in differentiated osteoblasts were also investigated and LLLT (685 nm, 2 J/cm^2^, 1–2 irradiations) promoted cell proliferation and viability along with expression of basic fibroblast growth factor (bFGF), insulin-like growth factor-I (IGF-I) and IGF-I (IGFBP3) receptor (21). In summary, stem cells tend to be less sensitive to a higher energy density than fibroblasts or keratinocytes.

Soleimani et al. (22) studied the influence of LLLT on the proliferation and differentiation of bone marrow-derived mesenchymal stem cells, induced to differentiate to either neuron or osteoblast. LLLT with wavelength of 810 nm (3 or 6 J/cm^2^) was used for neurons and 2 or 4 J/cm^2^ for osteoblasts at days 1, 3, and 5 of the differentiation processes. The analysis at day 7 of differentiation showed an increased proliferation but not when 6 J/cm^2^ was used, enhanced neurons and osteoblast differentiation at all energy densities used. Stein et al. (23) also reported that LLLT increased proliferation and differentiation of human osteoblast cell line along with significant increase in osteogenic markers including alkaline phosphatase (ALP), osteopontin and bone sialoprotein. These studies showed LLLT promoted cell differentiation in addition to cell proliferation, however differentiation was less sensitive to higher energy density.

In summary, findings from the laboratory studies showed that LLLT increased cell proliferation and supported the clinical findings of improved wound healing after LLLT. Another important observation from laboratory studies is the inverse relationship between power or energy density of LLLT to the cellular responses. Comparative studies demonstrated a diminished effect from a higher energy density, and further increases paradoxically resulted in inhibition of cell proliferation, migration, viability or ATP activity. Although LLLT promoted differentiation of stem cells, however, the response did not appear to have the sensitivity or the inverse relationship to higher energy density as with cell proliferation.

Wavelength of the LLLT used is an important factor on the efficacy of cellular response. Light in the red to the near infrared range of 600–1,070 nm was found to have the greatest effect on cell proliferation. This may be a physical phenomenon due to absorption or interference of light beyond this range. Light with shorter wavelength are absorbed by hemoglobin or melanin, and those with longer wavelength are absorbed by water, leaving only light within this range to reach the cells.

### Metabolism

Laboratory findings supported the theory that cytochrome c oxidase (CCO) is the site of action of LLLT as the main photoacceptor and photosignal transducer (43). CCO has four redox active metal centers (Cu_A_, Cu_B_, heme a and a_3_) with absorbency in the red to infrared wavelengths (600–1,070 nm). CCO is the terminal enzyme of the mitochondrial respiratory chain which mediates the transfer of electrons from cytochrome c to molecular oxygen by increasing mitochondrial potential and promotes ATP production (44). However, an alternative theory by Sommer (45) proposed that it is the reduction in the interfacial water layers (IWL) viscosity that mediates the increase in mitochondrial ATP synthesis. Thus, the LLLT-induced mechanism responsible for mitochondrial changes is still unresolved. Nevertheless, these mitochondrial changes could increase reactive oxygen species (ROS) release for inducing transcriptional changes and production of nuclear factor-κB (NF-κB) (46). NF-κB induces anti-apoptotic and pro-survival proteins along with cell proliferation and migration (47). Also, mitochondrial nitric oxide (NO) decreased after LLLT, and this in turn increased ATP production (6). In addition, calcium channel can be modulated by LLLT through ROS crosstalk, which influences cellular physiology and communication such as modifying local electrostatic fields and protein conformations (48).

Many laboratory studies demonstrated the influence of LLLT on the mitochondrial membrane potential, and changes in ROS, NO and intracellular calcium levels. Alexandratou et al. (24) found transient increase in mitochondrial membrane potential and intracellular calcium together with increases in ROS production and promotion of cytoplasmic alkalinization, demonstrating the multiple facets of LLLT-induced mitochondrial changes. LLLT was also found to alter mitochondrial morphology from a filamentous to a granular appearance with MTT assay, associated with an increase in cell proliferation of osteoblastic cells (25). A study by Chen et al. (26) showed that LLLT (810 nm) induced ROS and ATP production with activation of NF-κB in murine embryonic fibroblasts. Although these studies showed the influence of LLLT on various mitochondrial dynamics, the clinical implications remained unclear, and are simply considered as markers of LLLT activity.

The influence of energy density in LLLT-induced mitochondrial changes are very similar to the findings with cellular proliferation and favors a lower level of energy density. When the intracellular calcium profiles were compared between 3.6 and 12 J/cm^2^; 3.6 J/cm^2^ induced transient increase of intracellular calcium without any cell damage whereas 12 J/cm^2^ induced a linear increase of intracellular calcium and damage in cardiomyocytes (27). Zungu et al. (28) studied two different energy densities (632.8 nm, 5 and 16 J/cm^2^) and demonstrated that 5 J/cm^2^ increased mitochondrial membrane potential, intracellular calcium, ATP and cyclic adenosine monophosphate (cAMP), while 16 J/cm^2^ produced an opposite mitochondrial changes in the damaged fibroblast.

Findings with various wavelengths on mitochondrial changes are more complex. A study reported that 660 nm laser increased ROS production, the 800 nm laser reduced ROS production while 970 nm laser produced a moderate anti-oxidant activity in neutrophil polymorphonuclear granulocytes and keratinocytes (29). Zupin et al. (30) demonstrated that LLLT effect differs between 800 and 970 nm with 800 nm increased mitochondrial membrane potential and ROS production while 970 nm reduced calcium flow among dorsal root ganglion neurons. In contrast to energy density, the wavelength could influence mitochondrial changes in a less predictable manner. Further studies are warranted to unravel the complex kinetics of LLLT-induced mitochondrial changes.

Changes in mitochondrial energy states and cellular proliferation may have important clinical applications, particularly in tissue transplantation or ischemia reperfusion injury. LLLT may be used prophylactically to precondition both donor and recipient tissues to improve viability. The yield of skin cultures, stem cells or hematopoietic tissues used for transplantation may be improved with LLLT.

However, it is important to note the big difference in mitochondrial responses resulting from a small difference in wavelengths of <200 nm (29, 30). Some wavelengths (660, 800, and 970 nm) would have similarly boosted cell proliferation, however the mitochondrial responses were diametrically different. It remains unclear as to how these differences in the mitochondrial responses manifest clinically, and further research would be useful to determine the optimal wavelength to use.

### Angiogenesis

Apart from direct influence on cell proliferation and mitochondrial activity, LLLT also promotes angiogenesis. For the direct effect of LLLT on human umbilical vein endothelial cells (HUVEC), Terena et al. (31) showed red laser (660 nm, 1–20 J/cm^2^) could increase the viability and protein concentration of HUVEC from the second post-irradiation day. While infrared laser (780 nm, 1–20 J/cm^2^) generally reduced cell viability. For the investigations of LLLT-induced angiogenic protein changes, Cury et al. (49) investigated the influence of LLLT (660 or 780 nm, 30 or 40 J/cm^2^, seven consecutive days) on skin flap animal model, and found an increased angiogenesis in blood vessel counting with upregulation of angiogenesis markers VEGF and hypoxia-inducible factor-1α (HIF-1α), with downregulation of tissue remodeling marker matrix metalloproteinase-2 (MMP-2). Similar upregulation of VEGF by LLLT was also reported in granulosa cells (32). Studies demonstrated that LLLT (660 nm, 20 mW, 10 min) induced angiogenesis by a similar amount as exogenous VEGF (25 ng/ml) but significantly higher angiogenesis induction was found when exogenous VEGF and LLLT were combined (33). Thus, LLLT also potentiates the effect of VEGF on angiogenesis. Winter et al. (34) used light emitting diode (LED) at 635 nm, 80 mW/cm^2^, 24 J/cm^2^ to induce angiogenesis in HUVEC and chick embryo chorioallantoic membrane (CAM). The investigators found a significant increase in HUVEC proliferation and cellular network formation, with the CAM model showing a significant increase in the number of vessel junction. In summary, PBM promotes angiogenesis directly, but also by potentiating VEGF activity.

Promotion of angiogenesis may be an important factor to account for the improved wound healing with LLLT. Blood vessels are vulnerable to damage during chemo or radiotherapy, and tissue fibrosis ensues. There may be clinical applicability of using LLLT to promote angiogenesis and prevent or limit those collateral damages. LLLT may also be used to promote angiogenesis in transplantation and improve outcome. Since the effect of VEGF is enhanced synergistically by LLLT, further research is warranted to determine if adjunct use of VEGF clinically together with LLLT can be used to promote angiogenesis. However, angiogenesis and VEGF overexpression in tumor cells have long been regarded as poor prognostic indicators in oncology (50). Thus, caution should be exercised, and further studies are needed to examine the impact in oncology patients.

### Apoptosis

Apoptosis is programmed cell death and this process involves distinct morphological characteristics and signaling pathways with activation of caspases, which may be triggered by various stimulus including LLLT (51). The activation of apoptosis will subsequently lead to reduced cell proliferation and viability.

Frigo et al. (35) compared the effects of 2 energy densities using GaAlAs 660 nm laser (50 mW, 150 or 1,050 J/cm^2^, three consecutive daily irradiation) on human keloid fibroblasts and murine fibroblast 3T3. MTT assay showed an increased cell proliferation and reduced cell death from hypodiploid cell with LLLT of 150 J/cm^2^ LLLT. However, at 1,050 J/cm^2^ it decreased cell proliferation and increased the percentage of hypodiploid cells, which could be due to apoptosis or a reduced number of active dividing cells. Another study by Acauan et al. (52) investigated the influence of GaAlAs laser (830 nm, 71 or 135 J/cm^2^) on RT-induced morphological changes and caspase-3 in mice parotids given immediately before and 24 h after RT. Immunodetection showed both LLLT groups had a lower percentage of caspase-3 with improved preservation of the parotid gland, and better outcome at energy density of 135 J/cm^2^.

In summary, LLLT at lower energy range inhibits apoptosis but paradoxically promotes apoptosis at a higher energy range. However, current knowledge in this area is poor and the impact of modulation by LLLT in the healing and recovery processes is unclear. Furthermore, other cell death mechanisms including necrosis and autophagy may be influenced by LLLT. More investigation is needed to improve our understanding of LLLT-induced cell death and changes in cell viability, before this effect can be utilized clinically.

### Inflammation

RD, OM, and LE involve inflammatory responses in both early and chronic phases with the expression of inflammatory cytokines (Interleukins (IL), tumor necrosis factor-α (TNF-α) and polarization macrophages (3, 4, 53).

LLLT generally produces anti-inflammatory changes from a range of *in vivo* studies of brain, lung and spinal cord injuries (54), but the underlying cellular and molecular mechanisms are under debate. Studies on the inflammatory modulation by LLLT at cellular level were focused on the mediation of macrophage polarization and inflammatory cytokines expression. Macrophage is an important mediator of inflammation: M1 phenotype is the pro-inflammatory type for direct host-defense against pathogens, while M2 phenotype is involved in the resolution phase of inflammation and tissue repair (55). Silva et al. (36) reported that 660 nm but not 808 nm produced significant increases of NO, a marker of increased M1 macrophage phenotype expression in RAW 264.7 mouse monocyte or macrophage cell line. Another recent study investigated the influence of LLLT (660 and 780 nm, 17.5 J/cm^2^) on the M1 or M2a activated J774 macrophage cell line (37), and LLLT suppressed a range of macrophage-associated inflammatory proteins and pro-inflammatory cytokines in a time-dependent and wavelength-dependent manner. However, Fernandes et al. (38) reported the upregulation of pro-inflammatory cytokine IL-6 at 660 nm LLLT among M1 activated macrophages despite general suppression of other pro-inflammatory cytokines were observed. Thus, the differential macrophage responses demonstrated with different LLLT regimens may be incorporated into the anti-inflammatory treatment protocol to control both acute and chronic inflammation phases with the involvement of different cytokines.

LLLT also modulated inflammation of other cell types. Chu et al. (39) studied the effects of LLLT on inflammation-induced apoptosis protection of human endothelial cells. LLLT (660 nm, 11.46 J/cm^2^) attenuated TNF-α/cycloheximide-induced apoptosis with a reduction of caspase-3/7/8/9 along with an increased cell proliferation. Lee et al. (40) applied GaAlAs laser (660 nm, 8 J/cm^2^) on human periodontal ligament cells and reported inhibition of lipo-polysaccharide (LPS)-induced pro-inflammatory cytokines, such anti-inflammatory mechanism was possibly due to downregulation of NF-κB and upregulation of intracellular levels of cAMP. Maldaner et al. (13) reported similar anti-inflammatory effects of LLLT (660 nm, 3–8 J/cm^2^) on H_2_O_2_-induced inflammation of skin fibroblast cell line (HFF-1). LLLT at 4 J/cm^2^ partially reversed the activation of DNA oxidation, caspase 3/8, IL-1β/6 and IFN-γ induced by H_2_O_2_, with an increased level of anti-inflammatory IL-10. Similar to the findings by Fernandes on M1 macrophage, a report by Chen et al. on human umbilical cord mesenchymal stem cells found that LLLT (635 nm, 808 nm, 635 + 808 nm, 12 J/cm^2^, two daily treatments for 3 days) promoted the expressions of pro-inflammatory cytokines IL-1, IL-6 along with NF-κB (41), but this was also accompanied by an increase in cell proliferation.

Thus, the effects of LLLT on inflammation regulation are complex and may vary among cell types and LLLT regimens. Nevertheless, these studies suggested that LLLT regulates a wide range of inflammatory cytokines and macrophage polarization that are responsible for the development of RD, OM and LE.

## Differential Reactions of Normal Cell and Tumor Cell to LLLT

Since LLLT is an effective treatment option for oncology treatment-induced side effects including RD, OM, and LE (6), the responses of tumor cells to LLLT have been investigated and compared with normal cells. Bamps et al. (56) studied the post-LLLT (830 nm, 1 and 2 J/cm^2^) proliferation of head and neck squamous cell carcinoma (HNSCC) cell lines and reported an increased cell proliferation with expression of phosphor-protein kinase B (Akt), phospho-ERK and Ki67 gene markers implying an increased cancer aggressiveness. Rhee et al. (57) also showed that LLLT increased proliferation of anaplastic thyroid cancer cell line (FRO) and decreased transforming growth factor-β1 (TGF-β1), implying dysregulation of cell cycle. Moreover, activation of pAkt/HIF-1α could promote angiogenesis. Similarly, LLLT induced cell proliferation of osteosarcoma and lung carcinoma cells (58), and oral carcinoma cells (59). In addition, Zhang et al. (60) showed that LLLT irradiation of lower energy densities (≤25 J/cm^2^) promoted cervical cancer cell (HeLa) viability while impairment was found at a higher energy density (50 J/cm^2^). These reports demonstrated that LLLT does influence tumor cell proliferation in a similar way as normal cells.

There were also reports that demonstrated the difference in responses between cancer cells and normal cells to LLLT. Silva et al. (61) compared viability, proliferation and cell cycle phase changes between irradiated fibroblasts and breast cancer cells (MDA-MB-231), using 2.5 and 10 Gy ionizing radiation (IR) and LLLT (GaAlAs laser 660 nm, 30, 90, or 150 J/cm^2^) at 24 h post-IR. LLLT promoted cell viability and proliferation with reduction of senescence in fibroblasts. Tumor cells however had no significant LLLT-induced changes in cell viability, with reduction of proliferation and increase in senescence. Similar results was evaluated by Schalch et al. (62), using oral squamous cell carcinoma cell line with a reduction of cell viability and migration along with apoptosis activation by LLLT (660 and 780 nm). Differences in radiosensitivity of normal and tumor cells were studied by Barasch et al. (63) as the pre-IR LLLT of 4 J/cm^2^ reduced the IR killing effect in normal human lymphoblasts (TK6) and sensitized the killing effect in human leukemia cells (HL60). Schartinger et al. (10) compared LLLT-induced effects between human oral carcinoma cell line (SCC-25) and normal epithelial cells (BEAS-2B), and reported that although LLLT reduced cell proliferation in both types of cells, increased proportion of S-phase cells, decreased proportion of G1-phase cells and proapoptotic effect were found in the oral carcinoma cell line. Djavid et al. (64) used clonogenic assay to assess the pre-IR LLLT effects and found an inhibited colony development of ovarian cancer at 685 nm, while LLLT of 830 nm conferred radioprotective effects on normal fibroblasts.

Therefore, the response of cancer cells to LLLT vary and may differ considerably among different tumor types and laser settings. Caution should be exercised when using LLLT for treating radiotherapy-induced side effects as this may potentially promote tumor development.

## Bridging Laboratory Findings to Clinical Applications

Contemporary clinicians and researchers face difficulties in understanding safety issues of LLLT protocols for cancer patients because of the huge amount of biomedical information currently available in the scientific literature. The studies highlighted in this review provided the insight, evidence and justification for the use of PBM in medicine. A variety of cellular responses can be produced from a wide range of laser settings. However, the responses have the potential not only for clinical benefits but also for adverse consequences. The clinical implications from a number of these studies were far from being understood, and caution needs to be exercised, particularly with the use of LLLT in oncology.

In oncology, there may be circumstances when tumor cells will unavoidably receive overlapping irradiation from both RT and LLLT. Management of OM in patients with intraoral cancer receiving RT is an example. This will invariably raise the concerns whether LLLT may confer radioresistive protection to the tumor cells or encourage its spread by promoting cell proliferation and angiogenesis. Silveira et al. (65) reported controversial results on the proliferative effects of LLLT on head and neck squamous cell carcinomas in *in vitro* environments. Moreover, LLLT can also produce remote or systemic effects (66), mediated by release of growth factors or cytokines into the circulation (67). Nevertheless, LLLT is a significant advance in the management of OM, RD, LE and wound healing for oncology patients. Further studies should be performed to address these issues. LLLT may also selectively inhibit cancer cell proliferation (64) or enhance tumor cell killing with RT (63). This imply that LLLT may have a role as an adjuvant to RT to enhance cancer treatment and be incorporated into the RT treatment plan.

With the variety of tumor cell, differentiation, cell lines and responses to LLLT, designing clinical studies for oncology patients will be challenging. A wide knowledge gap exists between current laboratory findings and clinical applications of LLLT for oncology patients. This may be bridged by adopting a comprehensive approach to integrate clinical and laboratory studies together. Sonis et al. (68) demonstrated bridging cellular studies to clinical use in the management of OM in head and neck cancer patients. Laboratory studies on specimens taken from smears or biopsies during the clinical course of treatment will enable the cellular changes to be meaningfully paired to clinical changes and outcomes. Ambiguities arising from cellular changes previously observed in laboratory studies can be clarified from the findings of such integrated studies.

## Conclusion

LLLT influences a wide range of cellular activities in various cell types including normal and tumor cells. This review provides insights into the various cell responses which should be useful for establishment of LLLT protocols. Better designed studies to consolidate the laboratory and clinical effects of LLLT are needed to strengthen the role of LLLT. Since LLLT may induce tumor cell proliferation, caution should be exercised and further studies performed to establish the safety of LLLT for more extensive use in oncology.

## Author Contributions

ST and VT collected information and drafted the manuscript. SL supervised the study and edited the manuscript. SR, HL, and MK edited the manuscript. All authors contributed to the article and approved the submitted version.

## Conflict of Interest

The authors declare that the research was conducted in the absence of any commercial or financial relationships that could be construed as a potential conflict of interest.
